# Dynamical modelling of street protests using the Yellow Vest Movement and Khabarovsk as case studies

**DOI:** 10.1038/s41598-022-23917-z

**Published:** 2022-11-28

**Authors:** Amer Alsulami, Anton Glukhov, Maxim Shishlenin, Sergei Petrovskii

**Affiliations:** 1 School of Computing and Mathematical Sciences, University of Leicester, Leicester, UK; 2 Novosibirsk State University, Novosibirsk, Russia; 3 Sobolev Institute of Mathematics, Novosibirsk, Russia; 4 Peoples Friendship University of Russia (RUDN University), 6 Miklukho-Maklaya St, Moscow, Russia

**Keywords:** Mathematics and computing, Applied mathematics, Human behaviour

## Abstract

Social protests, in particular in the form of street protests, are a frequent phenomenon of modern world often making a significant disruptive effect on the society. Understanding the factors that can affect their duration and intensity is therefore an important problem. In this paper, we consider a mathematical model of protests dynamics describing how the number of protesters change with time. We apply the model to two events such as the Yellow Vest Movement 2018–2019 in France and Khabarovsk protests 2019–2020 in Russia. We show that in both cases our model provides a good description of the protests dynamics. We consider how the model parameters can be estimated by solving the inverse problem based on the available data on protesters number at different time. The analysis of parameter sensitivity then allows for determining which factor(s) may have the strongest effect on the protests dynamics.

## Introduction

Protests are an ubiquitous phenomenon of social dynamics that has often had significant impact on the human society^1–3^. A social protest (often taking the form of a street protest or a demonstration) is a public expression of objection or disapproval towards an idea or action, typically of a political or economic origin. Peaceful social protests are a normal component of a modern democratic society^4^. However, social protests, especially mass street protests are not always peaceful^5^. In fact, they are often accompanied by some forms of civil disorder and may sometimes involve violence, resulting in disruption of normal everyday life. One example of the latter is given by London 2011 riots^6^. Starting as an expression of disapproval to what was perceived in some parts of the society as police brutality (a black man suspected in criminal activities was killed by police during his attempted arrest), the street protests were soon hijacked by criminals, leading to looting and arson attacks in several cities across the UK, hence resulting in considerable and completely unjustified damage to public and private property. Protests can sometimes continue for a considerable time (e.g. months or even years) and may develop into large-scale riots or a revolution, resulting in significant political changes and far-reaching consequences; two well known historical examples are given by the Great French Revolution 1789–1799 (e.g. see^7^) and the Russian revolution 1905–1907^2,8^.

**Figure 1:**
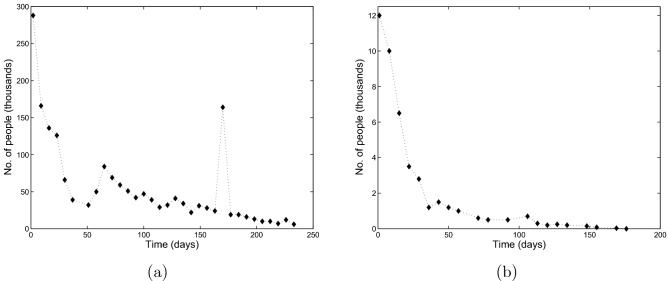
Number of people participating in the street protests vs time in the two motivating examples: (**a**) Yellow Vests Movement 2018–2019 (France), (**b**) Khabarovsk 2019–2020 (Russia). The dotted line is added for convenience of visualization only. For the YVM, the data were taken from Ref.^13^; for the Khabarovsk protests, the data were collected over 2020–2021 from relevant publications in the media^14^.

Understanding of factors that determine the duration and intensity (e.g. in terms of the number of people involved) of street protests is apparently a problem of high practical importance and indeed it has been a focus of research for a few decades^9–11^. While the phenomenon as a whole exhibits a wealth of different patterns and scenarios, there are some features that are repeatedly seen in several different street protests. In this paper, we focus on two events that arguably show a similar (albeit not identical) dynamics. The first selected event is the 2018–2019 Yellow Vest Movement (YVM) in France^12^; see Fig. 1a. The YVM took place as a response to the government decision to increase the fuel tax. The decision immediately became extremely unpopular as it was thought to lead to a considerable decrease in personal and family income. In order to express their disapproval, on Saturday November 17, 2018, almost 300,000 people took part in street protests across the country. The street actions then repeated every following Saturday for about a year. Although the number of participants was never as high as at the beginning of the movement (in fact, dropping about three times already over the first month), for several months it was fairly petrsistent, showing only a slow decay until autumn 2019 when it started decreasing fast to fully disappear by the end of the year. In the meantime (spring-summer 2019), the decrease in the number of YVM participants was so slow that it even caused a speculation that the movement would never disappear but instead might become a permanent feature of the French society (e.g. to give rise to a new political party).

For the second event, we consider the 2019–2020 street protests in Khabarovsk, Russia^15^; see Fig. 1b. On July 9, 2019, the Russian Federal Government, basing on the results of a long criminal investigation, arrested the Khabarovsk Region Governor Sergei Furgal. Since, on one hand, he was a very popular regional politician and, on the other hand, the level of trust to the Federal Government at that time was declining, a considerable part of the regional population met his arrest with dismay. Rumours were spreading that the real reason behind Furgal’s arrest was his tensions with the Federal Government rather than any criminal activity. As a result, willing to express their support to the popular politician, on Saturday July 11 a large number of people took part in a massive demonstration in the streets of Khabarovsk (several independent sources estimate that number as 12,000, although some other [unverified] sources put it as large as 80 thousand). The street protests continued on an approximately weekly basis for a rather long time afterwards, with the number of participants gradually decreasing to zero (see Fig. 1b).

We readily observe that the patterns shown in Fig. 1a,b exhibit similar features: a high peak at the beginning is followed by a gradual (not necessarily monotonous) decrease to small values, finally resulting in protests termination. Arguably, this similarity suggests a certain degree of universality of the observed pattern, in spite of the fact that the two events took place in countries with rather different political culture and traditions. Thus, one can hypothesize that the dependence of protesters number on time is determined by some generic features of social dynamics and/or human behaviour.

In spite of the apparent similarity between the two cases, there are certain differences too. One obvious feature of the YVM case is the trough in the participants number (*N*) as a function of time (*t*) observed in weeks 5–8 of the protests that was followed by the secondary peak in week 9. This is usually explained by the effect of public holidays, as weeks 5–7 overlapped with Christmas & New Year festivities. Such trough does not exist in the Khabarovsk case (although we observe traces of a local minimum around week 6). A closer look at the data reveals some more subtle differences too. Namely, while in case of Khabarovsk protests the protesters number shows a straightforward decay to zero, in the YVM case the shape of the graph of *N*(*t*) suggests that the system first converges to a certain positive ‘intermediate’ steady state value before the final convergence to zero. (In a recent study^16^, the latter type of dynamics was regarded as a ‘long transient’ caused by a ghost attractor^17,18^). Questions therefore arise to what extent the dynamics of YVM and Khabarovsk protests are actually similar (shaped by the same factors) and whether they can be descried by the same generic framework or model.

In this paper we attempt to look into the above issues to reveal what may be the differences between the corresponding system’s properties and parameter values that may lead to the observed variations in the *N*(*t*) pattern. Our goal is threefold. Firstly, we consider a generic model of street protests to show that, for different parameter values, it exhibits a variety of different dynamics, some of them qualitatively consistent with the data but others being rather different. The question thus arises how appropriate values of the model parameters can be found. Therefore, secondly, we consider an inverse problem to show how parameter values can be restored from the data. Thirdly and finally, we endeavour to reveal the differences and the commonalities between the two cases by interpreting the differences between the corresponding parameter values and their potential effect on the protests dynamics. One take-home message from our study is that, along with the strength of the protesters collective behaviour, the rate at which the collective effects build up are of crucial importance. In turn, it suggests that the non-uniformity in the distribution of behavioural thresholds is a factor significantly affecting protests intensity and duration.

## Mathematical model

### Model equations

Social protests can be studied quantitatively using a variety of research approaches, such as, for instance, a statistical analysis of the data on the protesters number^19^, including methods of geostatistics^20,21^, and the regression analysis of the participants social traits^22^. In our study, we use the approach based on the dynamical modelling where the variables describing the protest (e.g. the number of participants) evolve with time (and, more generally, in space) as a result of interactions between the individual protesters and protesters’ groups^6,9–11,23–31^. Namely, following a well established tradition^9,32,33^, we describe the dynamics of protests by regarding it, essentially, as the dynamics of human decision-making and behaviour. Protests can then be considered as as a certain ‘epidemics’^25^ (cf. “epidemics of bad behaviour”). In the nutshell, a ‘susceptible’ individual may decide to join the protests as a result of ‘catching’ this idea, either in person or via social media, from people who are already actively involved (hence regarded as ‘infected’). We mention here that a similar approach can also be used to study the dynamics (evolution) of opinion^9,34^, fashion^35^ and habit^36^.

**Figure 2:**
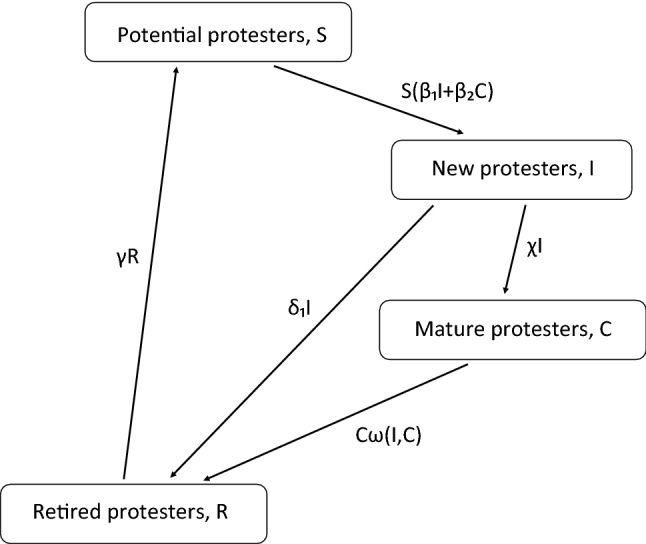
Flow-chart of the model of social protests. The boxes correspond to different groups, the arrows show transitions between the groups, the labels at the arrows show the transition rates. The active protesters $(I+C)$ are assumed to consist of newly engaged ones (*I*) and the experienced or mature ones (*C*), the former are recruited (as a result of the interaction with the active protesters) from a pool of potential protesters (*S*). Both new and experienced protesters can retire from the protests, e.g. after becoming tired or disillusioned by the protests, but can eventually join the pool of potential protesters again.

More specifically, we adopt the following model (cf.^16^). The whole population consists of four classes or ‘boxes’ (Fig. 2): *S* are potential protest participants, *I* are the new protesters who joined the movement recently, *C* are the experienced (mature) protesters and *R* are the former protesters who became disappointed or disillusioned and withdrew from the movement. Note that the explicit inclusion of experienced protesters into the consideration as a separate group is an essential new feature of our model. The existence of such ‘professional’ protesters is admitted in the literature as an inherent part of the movement society (e.g. see^37–39^) but their actual role in the protests dynamics remains under-investigated.

Correspondingly, the model consists of the following equations:

1 $$\begin{aligned} \frac{dS}{dt}&= {} -S(\beta _1 I+\beta _2 C) + \gamma R, \end{aligned}$$

2 $$\begin{aligned} \frac{dI}{dt}&= {} S(\beta _1 I+\beta _2 C) - \chi I - \delta _1 I, \end{aligned}$$

3 $$\begin{aligned} \frac{dC}{dt}&= \chi I - C\omega (I,C), \end{aligned}$$

4 $$\begin{aligned} \frac{dR}{dt}&= \delta _1 I + C\omega (I,C) - \gamma R. \end{aligned}$$

The number of street protesters at time *t* is therefore $N(t)=I(t)+C(t)$. Each term in the right-hand side of Eqs. (1)–(4) describe the transition between the groups, the coefficients having the meaning of the average transition rate per individual of the corresponding group. We mention here that, mathematically, system (1)–(4) is similar (albeit not identical) to the so called SEIR model of epidemic dynamics, e.g. see^40^.

Note that most of the rates in (1)–(4) are described by either linear or bilinear functions, the latter originating in the usual assumption that the probability of an average individual to change its ‘status’ (e.g. opinion) is proportionate to the frequency of contacts with people from different groups, which in turn is proportionate to the group size^9,23,34,40^. Linearity with regard to each of the variables in the bilinear product neglects any possible collective effects, in particular assuming that each participant in their decision-making acts strictly individually, i.e. not taking any account of what may be happening with other people in his group, e.g. their friends, colleagues, neighbours, etc. This, in turn, assumes a certain degree of atomization in the corresponding group.

While the above is likely to be true for new protesters, it may not necessarily be true for other groups. In particular, it seems reasonable to assume that for the mature protesters collective effects play a more significant role. Since experienced protesters, due to their definition in our model, have already spent some time (probably of considerable duration) participating together in street actions, it seems likely that they have established strong ties between themselves, in particular based on shared ingroup emotions^41,42^. Correspondingly, the opinion of others in their group is more likely to be important for them than it is in other groups, cf. “united we stand, divided we fall”^43,44^. Indeed, it is a well established observation that participants in a collective action learn from their experience and eventually shift their behaviour making the collective effects stronger^45^. As one result, a growing turnout in the street protest makes it more likely for an activist to engage^45,46^; in particular, this has been clearly shown to be true for left-leaning movements^38^.

Thus, collective effects in the dynamics of mature protesters are likely to be important and that should be taken into account in the per capita retirement rate of mature protesters as quantified by function $\omega (I,C)$ in Eq. (3). Therefore, based on the above heuristic arguments, we assume that the probability of a mature protester to retire is a decreasing function of the total number of protesters $N=I+C$ so that the per capita retirement rate $\omega (I,C)$ reaches its largest value when the number of protesters is small. More specifically, here we consider the following hypothetical parameterization:

5 $$\begin{aligned} \omega (I,C)= & {} \delta _2 +\delta _0\frac{C_0^n}{(I+C)^n+C_0^n}, \end{aligned}$$

where $\delta _0,\delta _2,C_0$ and *n* are parameters that determine the shape of the function in the following way. With an increase in $N=I+C$, the per capita transition rate $\omega$ monotonously decrease from $\delta _0+\delta _2$ to $\delta _2$. Thus, $\delta _0$ quantifies the strength of the collective effects. $C_0$ describes the ‘threshold’ protesters number when the collective effects become essential; in terms of the graph of $\omega$ as a function of *N*, that corresponds to the position of the curve’s inflection. Parameter *n* determines the steepness of the graph at the inflection point: the larger *n*, the more prominent the sigmoid shape of the graph is. For a more detailed argument resulting in functional form (5), see also the Supplementary Material.

With $\omega (I,C)$ chosen as in (5), system (1)–(4) takes the following form:

6 $$\begin{aligned} \frac{dS}{dt}&= {} -\beta _1 SI - \beta _2 SC + \gamma R, \end{aligned}$$

7 $$\begin{aligned} \frac{dI}{dt}&= {} \beta _1 SI+\beta _2 SC - \chi I - \delta _1 I, \end{aligned}$$

8 $$\begin{aligned} \frac{dC}{dt}&= \chi I - C\left( \delta _2 +\delta _0\frac{C_0^n}{(I+C)^n+C_0^n}\right) , \end{aligned}$$

9 $$\begin{aligned} \frac{dR}{dt}&= \delta _1 I + C\left( \delta _2 +\delta _0\frac{C_0^n}{(I+C)^n+C_0^n}\right) - \gamma R. \end{aligned}$$

In order to make the mathematical problem complete (making it the Cauchy problem), Eqs. (6)–(9) must be complemented with initial conditions, which we consider as follows:

10 $$\begin{aligned} S(0)=S_0, \qquad I(0)=I_0, \qquad C(0)=C_0, \qquad R(0)=R_0. \end{aligned}$$

In the rest of the paper, for the sake of technical simplicity we assume that the withdrawal rate for the experienced protesters is substantially smaller than that for the new ones, so that $\delta _2\ll \delta _1$. We also consider the recovery rate at which the retired protesters are joining the pool of potential protesters (quantified by $\gamma$) to be low, so that in most cases considered below $\gamma$ will be assumed to be either very small or zero.

### Models properties, steady states and their bifurcations

We first notice that system (1)–(4) has the conservation property:

11 $$\frac{dS}{dt}+\frac{dI}{dt}+\frac{dC}{dt}+\frac{dR}{dt}~=~\frac{d(S+I+C+R)}{dt} = 0,$$

so that, in our model, the total population size *P* remains constant during the whole duration of the protest movement:

12 $$\begin{aligned} S(t)+I(t)+C(t)+R(t) = P(t) = \text{ const } = P(0). \end{aligned}$$

Relating it to the real world, condition (12) reflects the observation that the protests duration rarely exceeds two or three years, more typically being on the scale of weeks or months. Meanwhile, the rate of the population change in European countries (due to the combined effect of the natural growth/mortality and the migration) almost never exceeds one percent for year, with very few exceptions^47^. Therefore, the rate of change in the number of protesters is much faster than that of the total population, and hence it is reasonable to consider the total population being constant. The mathematical meaning of (12) is that, in the system (6)–(9), only any three equations are independent and a fourth one can be replaced with Eq. (12).

We now investigate the existence and stability of steady states for the system (6)–(9). A steady state—say $({\bar{S}},{\bar{I}},{\bar{C}},{\bar{R}})$—arises as a solution of the corresponding algebraic system obtained from (6)–(9) when all time derivatives are zero:

13 $$\begin{aligned}&-\beta _1 SI - \beta _2 SC + \gamma R~=~0, \end{aligned}$$

14 $$\begin{aligned}&\beta _1 SI+\beta _2 SC - \chi I - \delta _1 I~=~0, \end{aligned}$$

15 $$\begin{aligned}&\chi I - C\left( \delta _2 +\delta _0\frac{C_0^n}{(I+C)^n+C_0^n}\right) ~=~0, \end{aligned}$$

16 $$\begin{aligned}&\delta _1 I + C\left( \delta _2 +\delta _0\frac{C_0^n}{(I+C)^n+C_0^n}\right) - \gamma R~=~0. \end{aligned}$$

It is readily seen that the trivial steady state (0, 0, 0, 0) exists for all parameter values but it is not by itself of much interest as it corresponds the trivial case where the population does not exist, i.e. $P(t)\equiv 0$.

For a positive steady state, i.e. for $({\bar{S}}>0,{\bar{I}}>0,{\bar{C}}>0,{\bar{R}}>0)$, system (13)–(16) cannot be solved analytically because of its algebraic complexity. Therefore, the steady states have to be found numerically. However, to solve numerically the nonlinear algebraic system (13)–(16) is a highly nontrivial problem. Instead, we solve the ODE system (6)–(9) using well established methods (e.g. by Runge-Kutta method using Matlab ode45 function); any stable steady state then emerges as a large-time stationary limit.

In order to find steady states numerically, we have to chose specific values for the model parameters. Parameter values relevant to the real-world protests dynamics will be discussed in the next section; here, in order to make a general insight into the properties of the model, we use some hypothetical parameter values as a starting point and then check how system’s properties may change with their variation.

Note that system (6)–(9) contains nine parameters. It is hardly doable to vary all of them. Instead, we choose two of them as ‘bifurcation parameters’ and vary them (each of them separately) in a certain range having other parameters fixed. Also, we additionally assume that the maximum retirement rate of the experienced protesters is equal to that of the new (novice) protesters. Thus, we begin with the following hypothetical parameter values: $\beta _1 = 0.0045,\ \beta _2 = 0.1832,\ \chi = 0.0203,\ n = 5,\ C_0 = 15,\ \delta _1=0.0762,\ \delta _2=0.002 , \ \delta _0 = \delta _1 - \delta _2=0.0742$.

**Figure 3:**
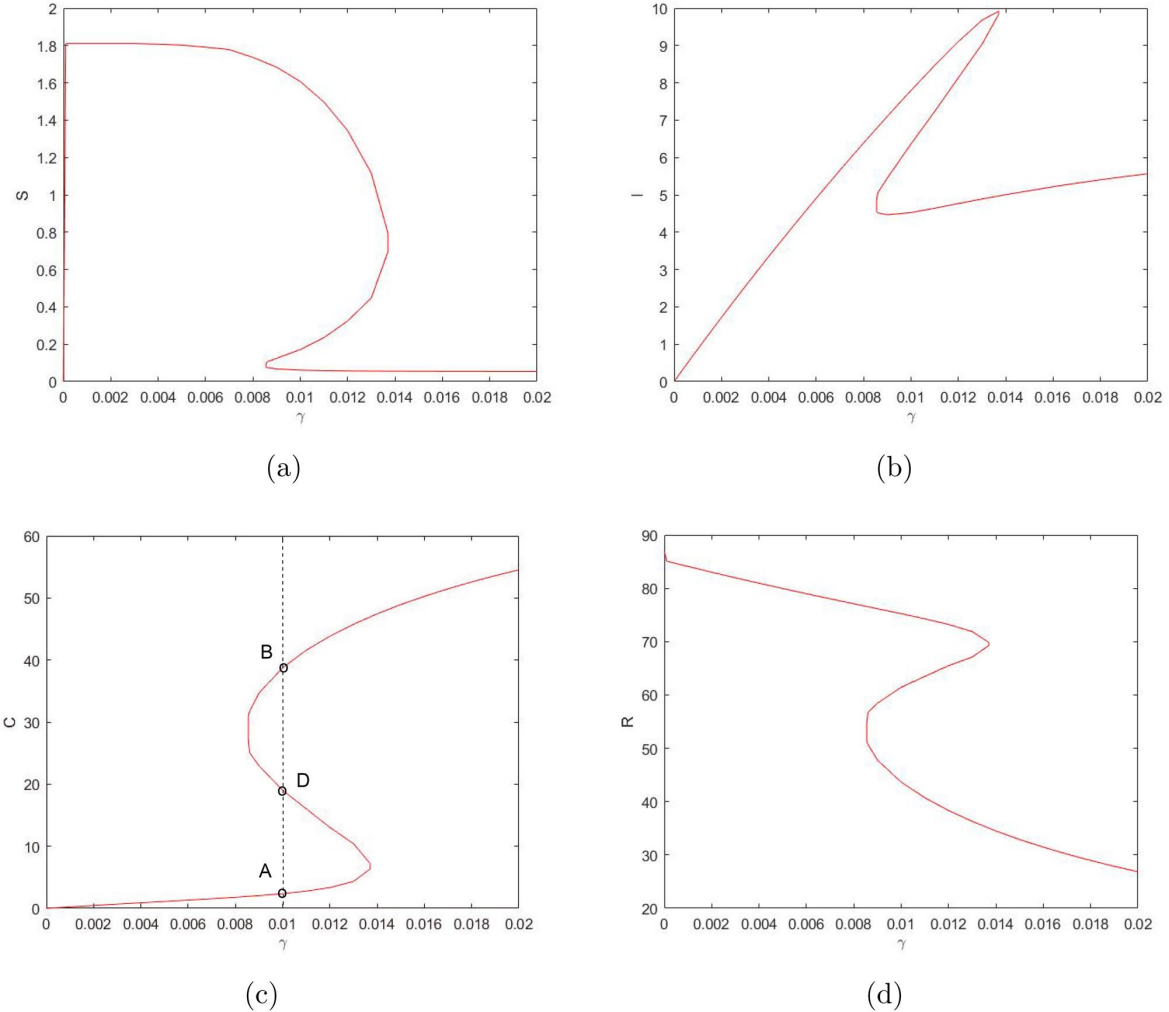
Positive steady state values of $S,\ I,\ C,\ \text {and}\ R$ (in (**a–d**), respectively) for the system (6)–(9) as a function of parameter $\gamma$. Other parameter values are given in the text. Here the lower and upper steady states are stable (cf. *A* and *B* in (**c**) as an example) and the ‘intermediate’ steady state (cf. *D*) is unstable. The sigmoidal shape of the curves indicates that the number of the steady states can change in response to a change in $\gamma$ (i.e. when the vertical line in (**c**) moves left or right).

Considering $\gamma$ as the bifurcation parameter, we obtain that the system can have up to three positive steady states (see Fig. 3). For sufficiently small values of $\gamma$, the system has only one steady state—say, steady state *A*. For sufficiently large values of $\gamma$, the system also has only one (but different) steady state—say, steady state *B*. But for intermediate values of $\gamma$, the system has three steady states, steady state *A*, steady state *B* and an intermediate steady state—say, *D*. Steady states *A* and *B* are stable nodes and steady state *D* is a saddle. When the value of $\gamma$ increases above a certain critical value, steady states *A* and *D* disappear in the saddle-node bifurcation, so that only steady state *B* remains feasible. When the value of $\gamma$ decreases below a critical value, steady states *B* and *D* disappear in the saddle-node bifurcation, so that only steady state *A* remains feasible. At $\gamma =0$, steady state *A* becomes a boundary steady state where ${\bar{I}}=0$ and ${\bar{C}}=0$ but ${\bar{S}}>0,~{\bar{R}}>0$, cf. Fig. 3.

**Figure 4:**
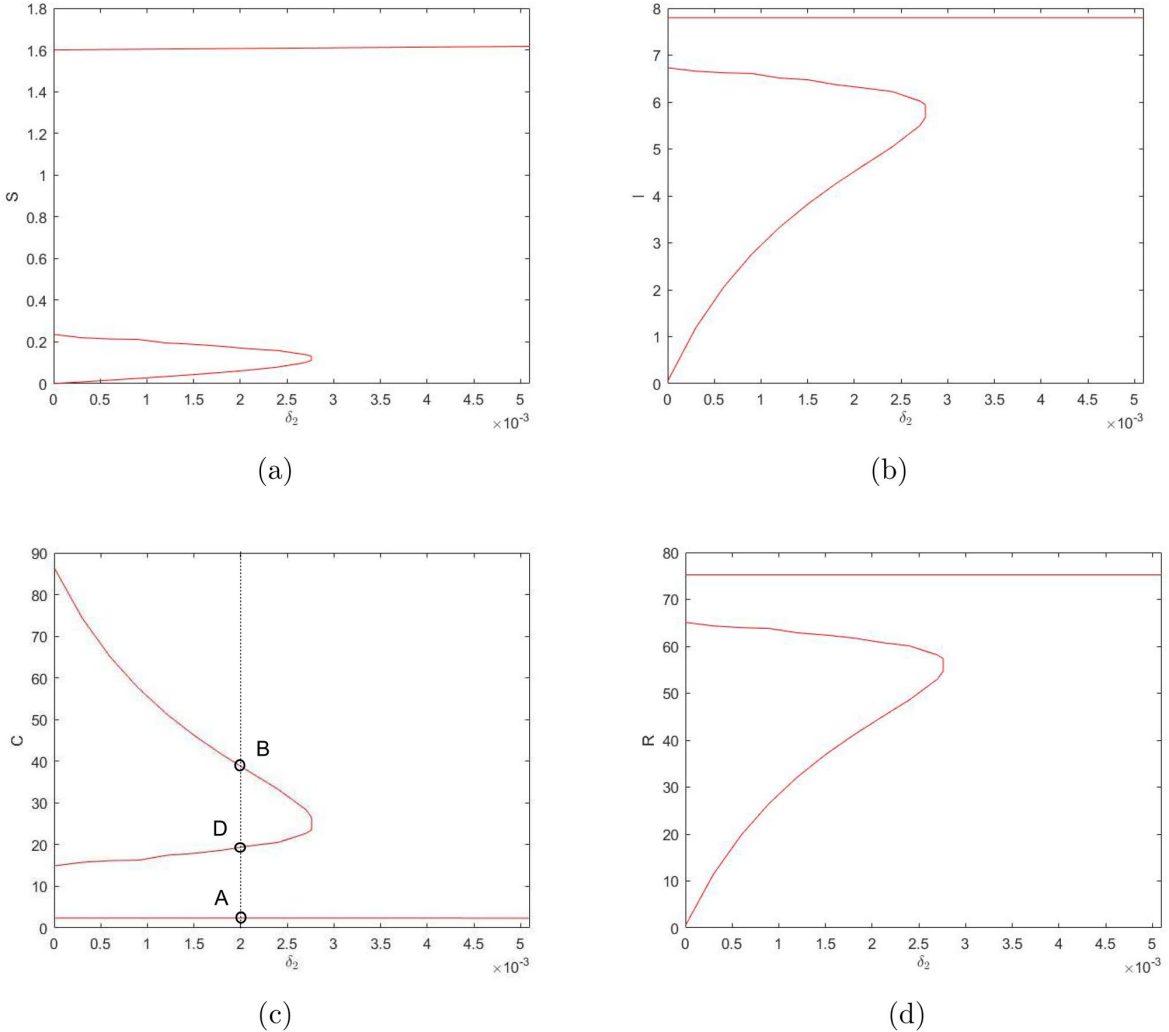
Positive steady state values of $S,\ I,\ C,\ \text {and}\ R$ (in (**a–d**), respectively) for the system (6)–(9) as a function of parameter $\delta _2$. Other parameter values are given in the text. The shape of the curves indicates that the number of the steady states can change in response to a change in $\delta _2$ (e.g. when the vertical line in (**c**) moves sufficiently far to the right).

Similarly, considering $\delta _2$ as the bifurcation parameter and taking into account its effect on system (6)–(9), we reveal the bifurcation structure of the system; see Fig. 4. It is readily seen that, for values of $\delta _2$ not too large, the system has three steady states: stable states *A* and *B* and unstable (saddle) state *D*. With an increase in $\delta _2$, states *B* and *D* disappear through the saddle-node bifurcation, so that for values of $\delta _2$ above a certain critical value there is only one positive steady state. On the contrary, a decrease in $\delta _2$ does not result in a bifurcation: the system exhibits bistability for any sufficiently small $\delta _2$. At $\delta _2=0$, state *B *becomes a boundary state where ${\bar{S}}={\bar{I}}={\bar{R}}=0$ but ${\bar{C}}>0$.

Due to the presence of multiple steady states, the dynamics of system (6)–(9) can be sensitive to the initial conditions. In particular, which stable steady state the system approaches in the long-time limit depends on where the dynamics started, i.e. what the initial system’s state is. Correspondingly, the choice of initial conditions deserves a discussion. In the two motivating examples for this study (see Fig. 1), one readily observes that the protests start with a large number of participants that would eventually decrease in the curse of time. Therefore, for the dynamics shown in Fig. 1, a sensible type of the initial condition should consist of a relatively large number of protesters $(I(0)+C(0))$. Assumptions about *S*(0) and *R*(0) are more flexible and may as well depend on a broader context (see below). Assuming that the protests do not have any preceding history, hence being a one-off event, the pool of potential protesters *S*(0)) should be sufficiently large and the number of retired protesters *R*(0) is small. With this arguments in mind, for the tentative numerical simulations in this section, we use the following set of values:

17 $$\begin{aligned} \text{ Set } \text{ A: } \qquad S(0)=60,\ I(0)=15,\ C(0)=12,\ R(0)=0. \end{aligned}$$

**Figure 5:**
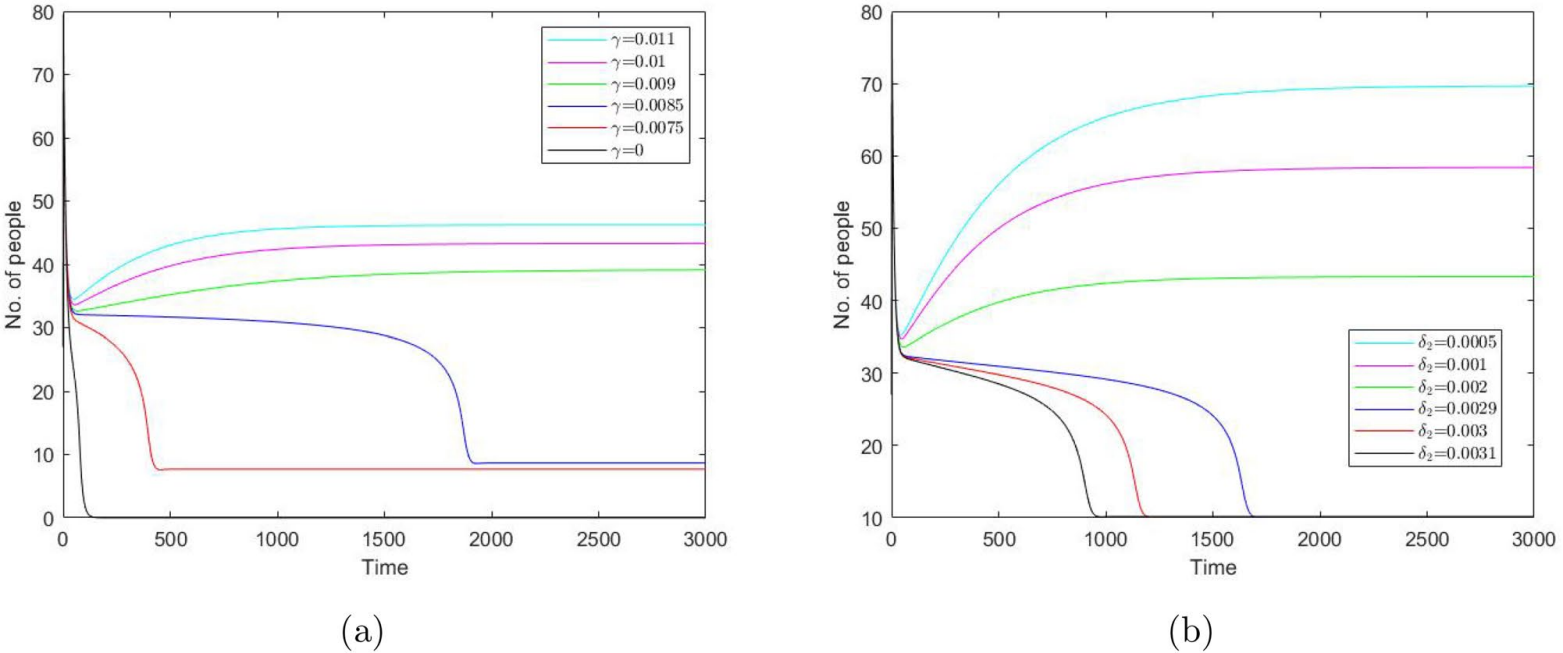
Number of protesters $I + C$ (in thousands) vs time (in days) obtained from Eqs. (6)–(9) for (**a**) different values of $\gamma$ and (**b**) for different values of $\delta _2$. Other parameter values are given in the text. The initial conditions are given by Set *A* (see Eqs. (17)). We readily observe that an increase in $\gamma$ or decrease in $\delta _2$ change the protest pattern making it persistent over time.

The system is solved numerically with hypothetical parameter values $\beta _1 = 0.0045,\ \beta _2 = 0.1832,\ \chi = 0.0203,\ n = 5,\ C_0 = 15,\ \delta _1=0.0762,\ \delta _2=0.002, \ \delta _0 = \delta _1 - \delta _2=0.0742$. The results are shown in Fig. 5. The shape of the curves is readily explained basing on the knowledge of the phase space structure. The inflection point of the black, magenta and blue curves, as well as the minimum for other three curves, is determined by the saddle point (cf. the unstable state *D* in Figs. 3, 4). Since a saddle point has an attracting manifold and a repelling manifold, for our choice of the initial condition the system first follows the attracting manifold, hence approaching the saddle point. Once the system comes sufficiently close to the saddle, the phase flow is averted and the system starts following the repelling manifold. Which part of the repelling manifold is followed and which steady state is approached as the large-time asymptotics (and hence whether the number of protesters increases or decreases with time) depends on the position of the initial state relative to the position of the manifolds, the latter depending on parameters $\gamma$ and $\delta _2$.

We also notice that the shape of at least some of the curves (e.g. the black one in Fig. 5a and the black, magenta and blue ones in Fig. 5b) are generally consistent with the generic shape of the protests data shown in Fig. 1: in all cases, they describe a monotonous decay of the protesters number with time, with the rate of decay varying with time.

**Figure 6:**
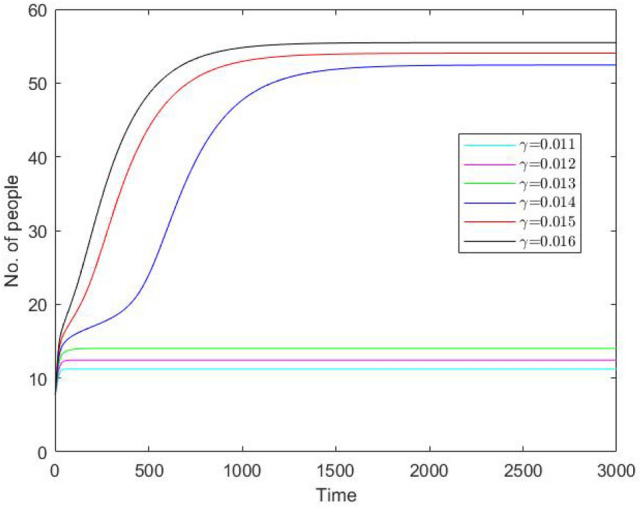
Number of protesters $I + C$ (in thousands) vs time (in days) obtained from Eqs. (6)–(9) for different values of $\gamma$ and the initial conditions as in Set *B* (see Eq. (18)). Other parameter values are given in the text. We readily observe that an increase in $\gamma$ tends to change the protest pattern resulting in a much larger number of protesters.

In order to demonstrate the potential of model (6)–(9) to describe a variety of dynamics, we also consider a different set of initial values corresponding to somewhat different societal conditions. Namely, we consider the case where the protests have a history. For instance, if other protests happened recently—where ‘recently’ means a timescale shorter than an average human lifetime, say within 20–30 years preceding the given event—then at least some (or, perhaps, many) of potential protesters might have previous street protests experience and hence should more appropriately be considered in class *R* rather than in *S*. Such ‘history of violence’ certainly exists in the case of France. In the case of Russia, this might seem less obvious; however, the events of early 1990s (the end of socialism and the collapse of the Soviet Union) could have a similar effect.

**Figure 7:**
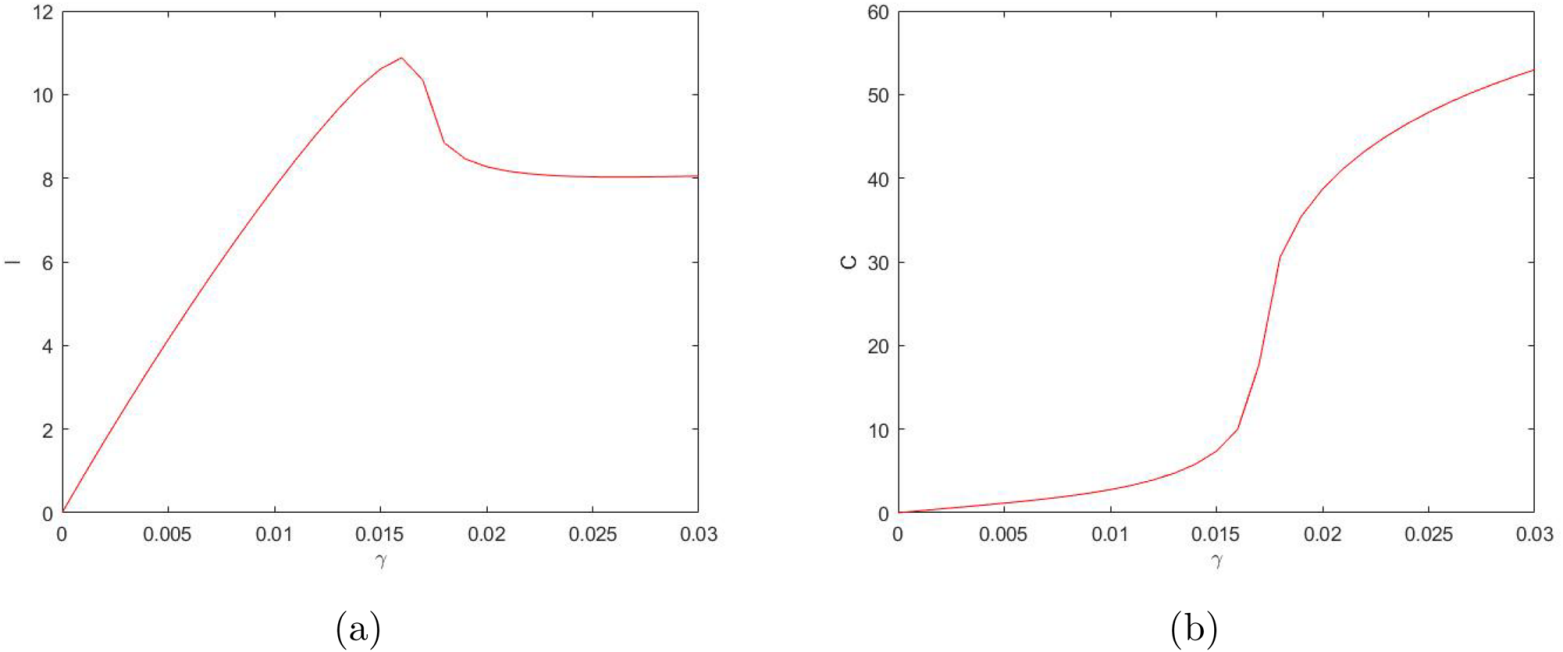
The steady state values of (**a**) *I* and (**b**) *C* for the system (6)–(9) obtained as a function of $\gamma$ for $n=3$. In this case, the number of steady states does not change with a change in $\gamma$ (no bifurcation occurs); there is always just one steady state. Other parameter values are the same as in Fig. 3.

Correspondingly, as an alternative set of the initial conditions we consider the following hypothetical values:

18 $$\begin{aligned} \text{ Set } \text{ B: } \qquad S(0)=1.2,\ I(0)=6,\ C(0)=1.8,\ R(0)=78. \end{aligned}$$

Solution of the system (6)–(9) obtained for the initial condition (18) and several different values of $\gamma$ (other parameters are the same as in Fig. 5a) are shown in Fig. 6. We readily observe that in this case the number of protesters does not show any tendency to decay. On the contrary, it increases and in the large-time limit stabilizes at some positive steady state value. This asymptotic value is small for values of $\gamma$ smaller than the critical value (for which the lower steady state *A* disappears, cf. Fig. 3c) but it can be large for larger, super-critical values of $\gamma$ where the only stable steady state is the upper steady state *B*.

One property of the system (6)–(9) that to a large extent shapes the properties of its solutions is bistability, i.e. the existence (for some intermediate values of $\gamma$ and sufficiently small $\delta _2$) of two positive stable steady states separated by an saddle point. An interesting question is therefore how the existence of the steady states and/or the dependence of the steady state values of the model variables *S*,  *I*,  *C* and *R* on the bifurcation parameters such as $\gamma$ and $\delta _2$ (see Figs. 3, 4) may change with a change in other parameters. In particular, *n* can be expected to have a significant effect, as it is the parameter that mostly determines the shape of the right-hand side of Eq. (8) (i.e. the retirement rate for the mature protesters). Indeed, our simulations show that a decrease in *n* can have a drastic effect on the shape of the curves by changing the *S*-shaped dependence (characteristic for bistability) to a single-valued function, hence eliminating bistability. As an example, Fig. 7 shows the dependence of *I* and *C* on $\gamma$ obtained for $n=3$; curves for *S* and *R* have similar shape (not shown here for the sake of brevity). For other parameter values fixed as in Fig. 3, this change in the shape of the curves from *S*-shape to a single-valued curve occurs for $n=n_{cr}\approx 3.5$.

**Figure 8:**
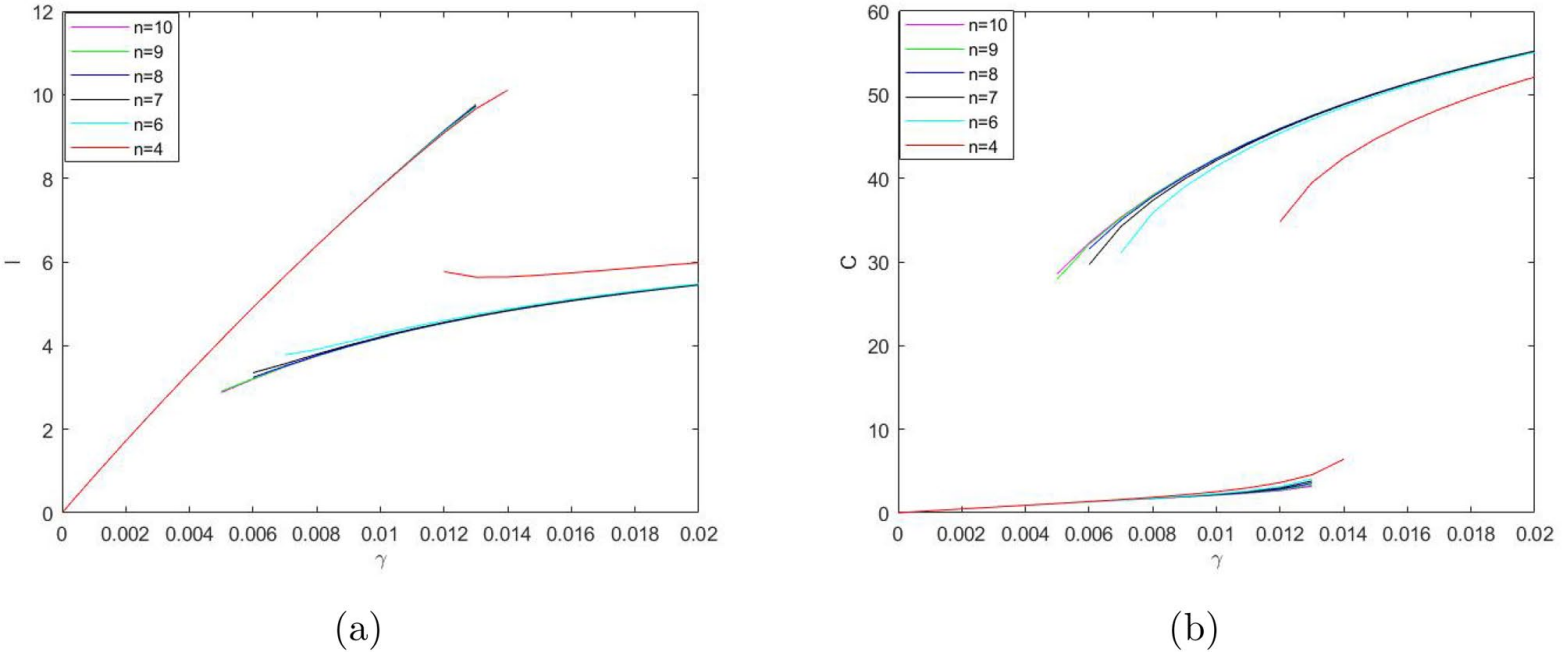
The two stable branches (the unstable branch is not shown for the sake of visualization clarity) of the steady state dependence on $\gamma$ obtained for different values of *n* between 4 and 10; (**a**) for *I*, (**b**) for *C*. Thus, in all cases the system exhibits bistability. Other parameter values are the same as in Fig. 3.

**Figure 9:**
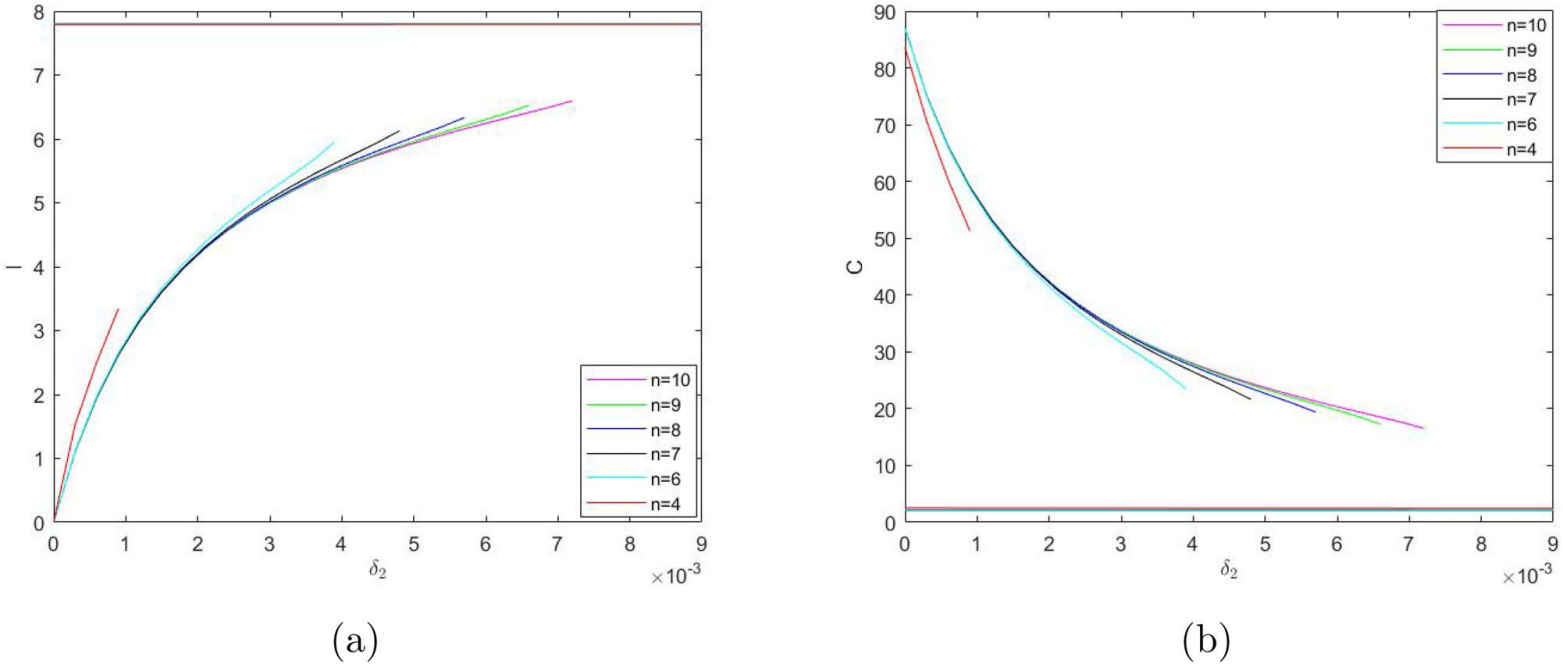
The two stable branches (the unstable branch is not shown for the sake of visualization clarity) of the steady state dependence on $\delta _2$ obtained for different values of *n* between 4 and 10; (**a**) for *I*, (**b**) for *C*. Thus, in all cases the system exhibits bistability. Other parameter values are the same as in Fig. 4.

On the contrary, an increase in *n* has relatively little effect on the shape of the curves. Figure 8 shows the two stable branches of the steady state dependence on $\gamma$ obtained for different values of *n* between 4 and 10. We readily observe that an increase in *n* does not eliminate bistability but slightly extends the range of $\gamma$ where bistability occurs.

A change in *n* has a similar effect on the dependence of the steady state values on $\delta _2$ (cf. Fig. 4). An increase in *n* extends the bistability range but does not bring any qualitative change in the shape of the curves, see Fig. 9. With a decrease in *n*, the tongue shaped curve for the stable-unstable pair shrinks to the left disappearing at $n_{cr}\approx 3.5$. For $n<n_{cr}$, the dependence of the steady state values on $\delta _2$ is given by a single-valued function showing only very weak dependence on $\delta _2$ (not shown here for the sake of brevity), similar in shape to the upper and lower lines in Fig. 9a,b. respectively.

## Inverse problem

Now we will attempt to compare the predictions of model (6)–(9) with the available data on the two protest movements, i.e. YVM 2018-19 in France and Khabarovsk 2019–2020 in Russia. Since the properties of the model can be rather different in different parameter ranges, the choice of parameter values is a crucial issue. Once the data are available, the search for parameters value that would provide the ‘best possible’ (in some sense) fitting of the data is a classical inverse problem. A standard approach to this is to minimize the difference between the (discrete) data and the (continuous) solution of the model—i.e., the ‘distance’ (in terms of a certain functional norm) between the two sets. Note that data on the relative abundance of the amateur protesters and the experienced protesters are not available; therefore, in comparing the model predictions to the data on the number of protesters we use the sum $I(t)+C(t)$.

A more rigorous formulation of the inverse problem is as follows. Let us suppose that we have the following set of data:

19 $$\begin{aligned} I(t_k) + C(t_k)=f_k, \qquad k=1,\ldots ,K, \end{aligned}$$

where *K* is the number of available data points.

Let us further suppose that some of the parameters of the direct problem (6)–(10) are unknown. Note that these parameters are not only the coefficients in Eqs. (6)–(9) but can also include the initial data (10). Later we will specify which parameters we attempt to recover. The inverse problem consists in finding the vector of unknown parameters $\mathbf{{q}}=(q_1,\ldots ,q_M)$ in (6)–(10) (where *M* is the number of unknown parameters) by relating the solution of the direct problem to the known data (19).

In a generic operator form, the inverse problem (6)–(10) and (19) can be formulated as follows:

20 $$\begin{aligned} A(\mathbf{{q}})=f \quad \Rightarrow \quad \mathbf{{q}}=A^{-1}f, \end{aligned}$$

where operator *A* maps ${\mathbb {R}}^M$ to ${\mathbb {R}}^K$ and $A^{-1}$ is the inverse operator.

We now reduce the solution of the inverse problem (20) to the minimization of a certain functional. Note that the distance between the data and a relevant solution of the model can be defined differently depending on the choice of the norm in the corresponding functional space. In turn, a different choice of norm can result in somewhat different parameter values. It is therefore desirable to solve the inverse problem with with more than one functional in order to reveal how sensitive the obtained parameter values are to the choice of functional.

Correspondingly, in the simulations below, we use the following two functionals:

21 $$\begin{aligned} F_1(\mathbf{{q}}) = ||A(\mathbf{{q}})-f ||_{l_1} = \sum \limits _{k=1}^{K} |I(t_k;\mathbf{{q}}) + C(t_k;\mathbf{{q}}) - f_k|, \quad \ \min _\mathbf{{q}}F_1(\mathbf{{q}})\rightarrow \mathbf{{q}}^{(1)}, \end{aligned}$$

and

22 $$\begin{aligned} F_2(\mathbf{{q}}) = ||A(\mathbf{{q}})-f ||^2_{l_2} = \sum \limits _{k=1}^{K} \left( I(t_k;\mathbf{{q}}) + C(t_k;\mathbf{{q}}) - f_k\right) ^2, \quad \ \min _\mathbf{{q}}F_2(\mathbf{{q}})\rightarrow \mathbf{{q}}^{(2)}, \end{aligned}$$

where $I(t_k;\mathbf{{q}})$, $C(t_k;\mathbf{{q}})$ means the direct problem solution for a given parameter set $\mathbf{{q}}$. Note that (22) corresponds to the $R^2$ method routinely used in statistics.

Minimization of the functionals can be performed by applying the global optimization methods (genetic, particle swarm optimization, differential evolution and others), gradient method or Newton type methods. In order to increase the efficiency, a combination of methods is often used, for example, global optimization methods are first used to determine the intervals of the minimum functional, and then gradient type methods are used to refine the parameters^48^. Also, adding a priori information about the inverse problem solution significantly decrease the number of iterations^49^. We assume that unknown parameters do not change during social protest and we use the method of differential evolution^50^.

### The Yellow Vest Movement

We first consider the case of the Yellow Vest Movement where we disregard the apparent outlier in the number of protesters in the 166th day of the protests. For the initial conditions, we assume that the street protest participants consist predominantly from people aged between 20 and 64 years. According to statistics for 2020, the size of this age group of the French population is about 55.5 million people, which we use as the cumulative initial condition, i.e. for $S(0) + I(0) + C(0) + R(0)$. Assuming that γ = 0 (for the reasons discussed above), the  results of the inverse problem solution are shown in Table 1. It is readily seen that, for both functionals (21) and (22), the method returns close values for *n*, $C_0$ and $\delta _1$ while the values for other parameters differ 2–3 times.

**Table 1:** Parameter values obtained from solving the inverse problem using the functional $F_1$ (Eq. (21)) for the second column (parameter set $\mathbf{{q}}^{(1)}$) and $F_2$ (Eq. (22)) for the third column (parameter set $\mathbf{{q}}^{(2)}$).

	Parameter set $\mathbf{{q}}^{(1)}$	Parameter set $\mathbf{{q}}^{(2)}$
$\beta _1$	0.0103 $\times 10^{-9}$	0.0194 $\times 10^{-9}$
$\beta _2$	0.2393 $\times 10^{-6}$	0.1045 $\times 10^{-6}$
$\chi$	0.0085 $\times 10^{-2}$	0.0163 $\times 10^{-2}$
*n*	1.5885	1.4993
$C_0$	22966	27975
$\delta _1$	0.0783	0.0684
$\delta _2$	0.5362 $\times 10^{-3}$	0.2167 $\times 10^{-3}$
$F_1$	246.56 $\times 10^{3}$	250.66 $\times 10^{3}$
$F_2$	5441.3 $\times 10^{6}$	4712.57 $\times 10^{6}$

The initial conditions are used as $S(0) = 55,212,000$ and $I(0) = 288,000$, $C(0) = R(0) = 0$. We readily observe that several parameters (in particular, $\beta _1,~\chi ,~n,~C_0$ and $\delta _1$) have close values, which indicates the robustness of parameter estimation to the choice of the functional.

**Figure 10:**
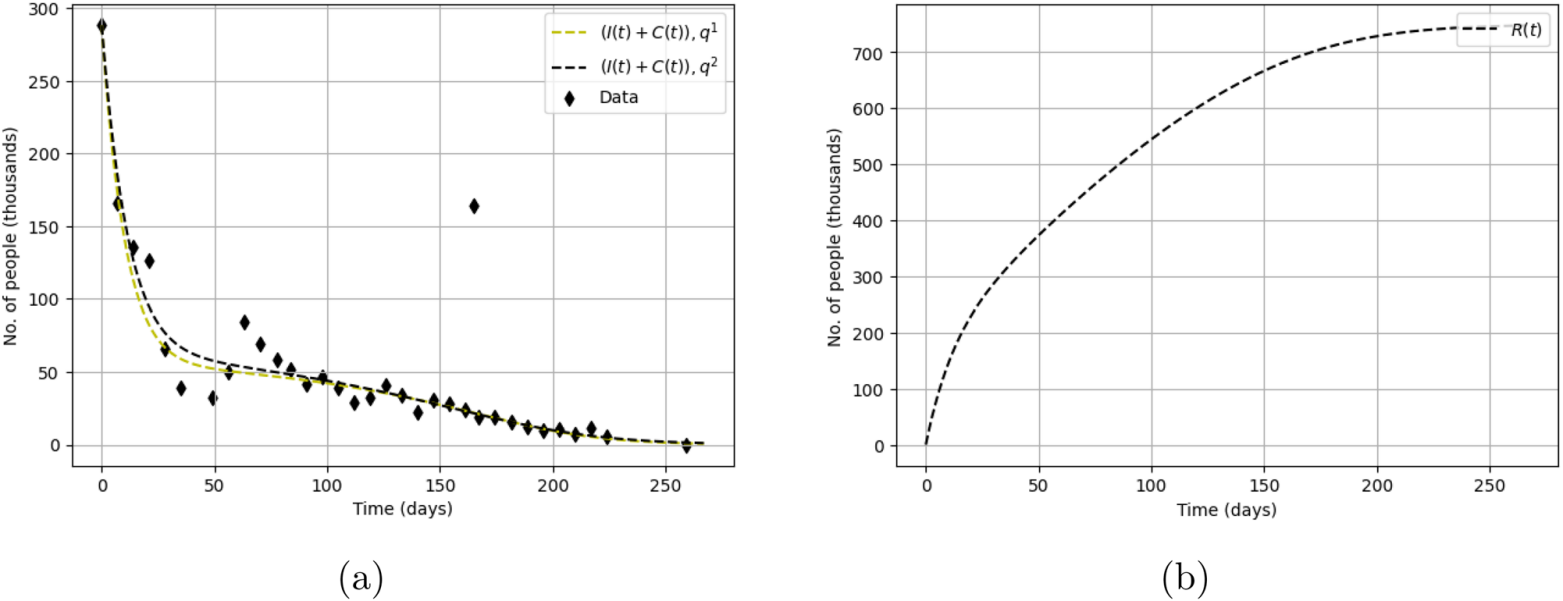
(**a**) Solution of the model (6)–(9) obtained for parameter sets $\mathbf{{q}}^{(1)}$ and $\mathbf{{q}}^{(2)}$ (cf. Table 1) shown in comparison with the data on the Yellow Vest Movement. The initial conditions are chosen as $S(0) = 55,212,000$, $I(0) = 288,000$ and $C(0)=R(0)=0$. (**b**) Dynamics of one of the ‘hidden variables’: the number of retired protesters *R* vs time as predicted by the model (6)–(9) obtained for parameter set $\mathbf{{q}}^{(2)}$.

Solutions of the model (6)–(9) obtained for the parameters from Table 1 are shown in Fig. 10a. We readily observe that, for both parameter sets, the solution of the model is in a good agreement with the data. In fact, the two solutions appear to be almost indistinguishable from each other, which apparently suggests that the solution of the direct problem is sufficiently robust to parameter values. In both cases, the solution describes well the first stage of the protests (the fast decay from the initial value over the first 3 weeks) and the long tail of the distribution during the later stage. Note that the solution has a slightly sigmoidal shape at the end of the tail, which can be the effect of a ghost attractor in the phase space of the system^16,18^. The solution does not describe the non-monotonous dynamics of protests during weeks 4–9. As was discussed in the introduction, this non-monotonous behaviour is not necessarily an innate property of the protests dynamics and might be explained by the effect of some external factors (not included into the model).

Note that our model allows to gain an insight into the ‘hidden’ properties of the protests dynamics, i.e. the variables that are not directly available from the data. As one example, Fig. 10b shows the number of retired protesters vs time predicting the final value on the order of 750,000 people. Given the massive scale of the YVM, especially over the first several weeks, this estimate seems to be consistent the reality.

### Khabarovsk protests

We now apply the same method to the data on Khabarovsk protests. The results of the inverse problem solution are shown in Table 2. We readily observe that, as well as in the previous case, both functionals produce similar parameter values (albeit quite different from the YVM case), in particular almost coinciding for $\beta _1$ and $\delta _1$. For parameters $\chi$, $C_0$ and $\delta _2$ the difference is within 40%; the values for other parameters differ about 2–3 times.

**Table 2:** Parameter values obtained from the solution of the inverse problem for the data on the Khabarovsk protests: $\mathbf{{q}}^{(1)}$ (second column) by using functional (21), $\mathbf{{q}}^{(2)}$ (third column) by using functional (22).

	Parameter set $\mathbf{{q}}^{(1)}$	Parameter set $\mathbf{{q}}^{(2)}$
$\beta _1$	0.1173 $\times 10^{-5}$	0.1167 $\times 10^{-5}$
$\beta _2$	0.4309 $\times 10^{-3}$	0.2348 $\times 10^{-3}$
$\chi$	0.1014 $\times 10^{-4}$	0.1408 $\times 10^{-4}$
*n*	9.473	3.6982
$C_0$	105.3403	75.5561
$\delta _1$	0.7102	0.7044
$\delta _2$	0.0279	0.022
$F_1$	2165	2422
$F_2$	837635	688192

The initial conditions are used as $S(0) = 604,000$ and $I(0) = 12,000$, $C(0) = R(0) = 0$ where $S(0) + I(0) + C(0) + R(0)=616,000$ is the population of Khabarovsk at 2020. We readily observe that several parameters (in particular, $\beta _1,~\chi ,~C_0,~\delta _1$ and $\delta _2$) have close values, which indicates the robustness of parameter estimation to the choice of the functional.

**Figure 11:**
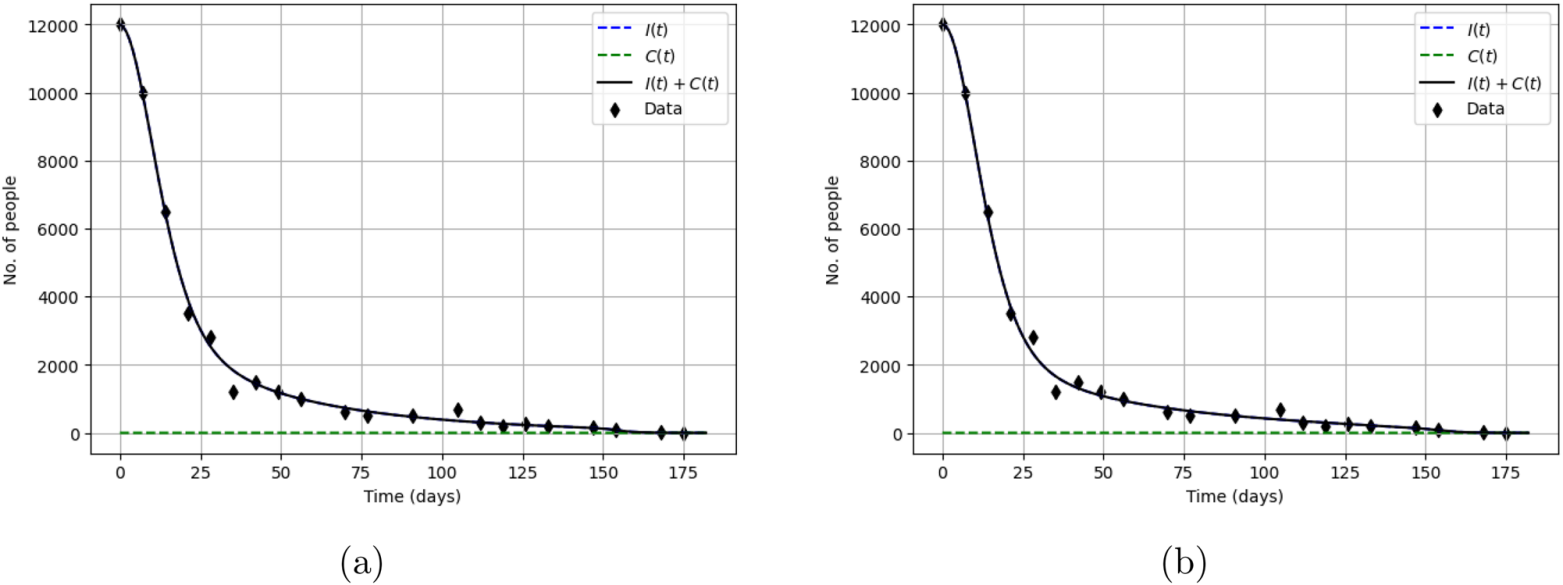
Solutions of the model (6)–(9) obtained for parameter sets (**a**) $\mathbf{{q}}^{(1)}$ and (**b**) $\mathbf{{q}}^{(2)}$ (see Table 2) shown in comparison with the data on the Khabarovsk protests. We readily observe that, for the two parameter sets, the solution fits the data equally well. We therefore conclude that the accuracy of the model to describe the data is robust with regard to the choice of functional.

Solutions of the model (6)–(9) obtained for the parameters in Table 2 are shown in Fig. 11, panels (a) and (b) for $\mathbf{{q}}^{(1)}$ and $\mathbf{{q}}^{(2)}$, respectively. In both cases, the solution describes the data quite well, apart from small deviations that may be of purely stochastic origin. As well as in the above case of YVM, a closer look reveals that the curves have a slightly sigmoidal shape at the end of the tail, hence suggesting, as well as in the YVM case, the effect of a ghost attractor^16,18^.

## Sensitivity analysis

### Parameters identifiability

We now estimate the identifiability of unknown parameters using sensitivity analysis. Consider the parameter vector as $\mathbf{{q}} = (\beta _1, \beta _2, \chi , n, C_0, \delta _1, \delta _2)$. In order to analyze the sensitivity of these seven parameters of our mathematical model (6)–(9), we follow the approach previously used in Ref.^51^ for SEIR-HCD pandemic model.

Sensitivity analysis methods are based on the construction of a sensitivity matrix^52^. Let $t_1 \le t_2 \le \ldots \le t_N$ be fixed time points at which a certain quantity *f* is ‘measured’ (in our case, the number of protests participants at a given date). Then the coefficients of the sensitivity matrix for the parameter vector $\mathbf{{q}}=(q_1,\ldots ,q_J)$ are calculated as follows:

23 $$\begin{aligned} \displaystyle s_{ij} = q_{j}\frac{\partial f(t_i, q) }{\partial q_j}, \quad j=1,\ldots ,J, \end{aligned}$$

(in our case $J=7$) where $i = 1,\ldots ,N$ is the number of available data points (measurements), $q_j$ is the *j*th component of the parameter vector, $I(t_i, q) + C(t_i, q) = f(t_i, q)$ are the inverse problem data. The quality of the parameter values estimation therefore depends on the number of available data points.

Since sensitivity analysis considers the property of the model rather than model’s specific application, in order to gain an insight into the sensitivity of model (6)–(9), it is sufficient to consider one set of data (i.e. one case study). Correspondingly, below we address the issue of sensitivity using the YVM data, as the corresponding dataset has more data points.

Let us consider the semi-relative sensitivity function for the solution vector $\mathbf{{x}} = (S, I, C, R)$ to calculate the coefficients of the sensitivity matrix:

24 $$\begin{aligned} \displaystyle s_{q_j}(t) = q_{j}^*\frac{\partial \mathbf{{x}}(t)}{\partial q_j}, \qquad j = 1,\ldots ,7. \end{aligned}$$

**Figure 12:**
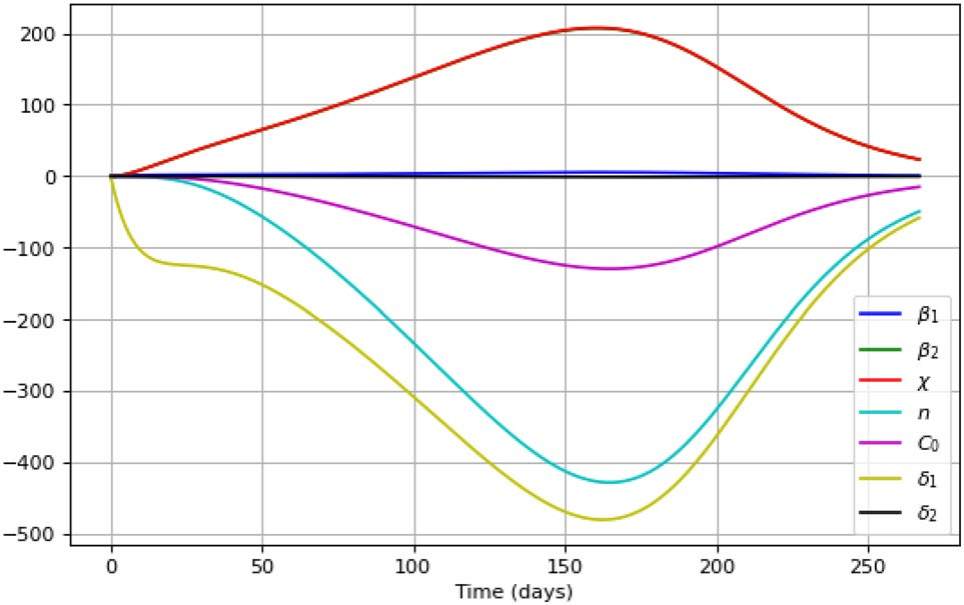
Sensitivity functions for all parameters of the system (6)–(9) obtained for the ‘measured’ variable $(I+C)$, with simulations performed for parameter set $\mathbf{{q}}^{(2)}$ (cf. Table 1).

Figure 12 shows the components of the sensitivity matrix as functions of time obtained for parameter set $\mathbf{{q}}^{(2)}$ (cf. Table 1). We readily observe that the quality of parameter value estimation for $\beta _1$ and $\delta _2$ shows only a weak dependence on the amount of data on the number of protests participants $(I+C)$, while the values of the remaining parameters exhibit a significant response to the amount of data. We also notice that, interestingly, the graphs of sensitivity functions for parameters $\beta _2$ and $\chi$ nearly coincide.

Now we will investigate the identifiability of the model parameters. A parameter is identifiable if it is in principle possible to obtain its the true value when an infinite amount of data (number of observations) is available. In order to investigate the parameters’ identifiability, we apply the orthogonal method^53^. The method requires a certain parameter set as a starting point, for which we will use set $\mathbf{{q}}^{(2)}$ (cf. Table 1).

The idea of the orthogonal method is to consider the (almost) linear dependence between the columns of the sensitivity matrix (23). In this way, the sensitivity of the response of the system to variations of parameter values is investigated, as well as possible inter-dependence between some of the parameters with respect to their influence on the system’s solution.

In practice, the orthogonal method consists of the following algorithm^53,54^:

1. Define an array of indexes of identifiable parameters $I = \varnothing$ and an array of unidentifiable parameters $U = \{1,\ldots ,7\}$.
2. Select the column *k* of the matrix *S* with the largest sum of squares of elements, add it to the matrix *E*, remove it from *S*. We add the index *k* to *I*, remove it from *U*.
3. If $U = \varnothing$, then stop, else go to step 4.
4. Find orthogonal projections of vectors from *S* to the subspace of *E* and construct a matrix of perpendiculars $S^\perp$ to the matrix *S* consisting of *h* remaining columns:
  $$\begin{aligned} S_p^\perp = S_p - S_p^{proj}, \qquad S_p^{proj} = \sum \limits _{i=1}^{7 - h}\frac{(S_p, E_i)}{(E_i, E_i)}E_i. \end{aligned}$$
  Here $$E = (E_1,\ldots , E_{7-h})$$, $$p = 1,\ldots ,h$$.
5. Select the column *k* of the matrix $S^\perp$ with the largest sum of squares of elements, add the index *k* to *I* and remove from *U*. Correspondingly, remove the corresponding column from *S* and add it to the matrix *E*. Go to step 3.

**Figure 13:**
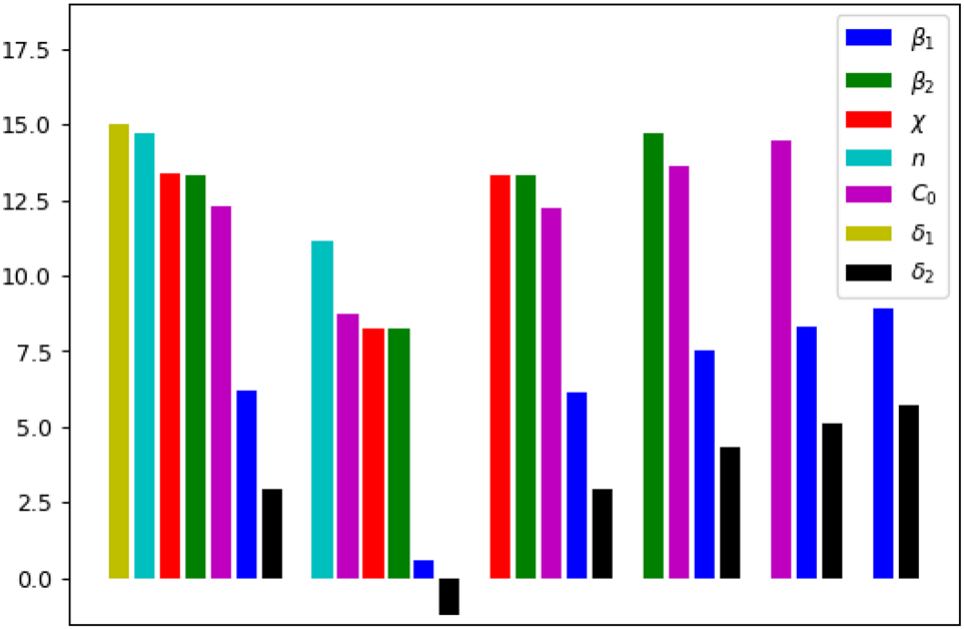
The norm of the perpendiculars (in logarithmic scale) calculated at each iteration of the orthogonal method. The horizontal axis shows the succession of algorithm’s iterations (six altogether). Note that, after six steps, only parameters $\delta _1$ and $\delta _2$ survive, which indicates their strong inter-dependence.

The results of the parameter identifiability analysis for our model (6)–(9) at various iterations of the orthogonal method are presented on Fig. 13. A sequence of parameters (from the most to the least identifiable) is obtained: $\delta _1$, *n*, $\chi$, $\beta _2$, $C_0$, $\beta _1$, $\delta _2$. Note the strong dependence between the parameters $\delta _1$ and $\delta _2$. It means that, while the effect of other parameter on the model’s predictions is unique, the influence of $\delta _2$ on the solution of the system can be partially compensated by changes in parameter $\delta _1$.

### Data uncertainty

In the above, we have implicitly assumed that the data—i.e. the number of protesters at any given date—are precise. This is, however, not always true. Firstly, the officially available data are usually rounded up to the nearest thousand or nearest hundred. Secondly, and perhaps more importantly, the way the data are collected is far from accurate: in fact, the number of protesters at any particular date is typically an estimate rather than a direct count, especially when the number is large. Thirdly, the number of protesters is the kind of information that is often politically loaded and hence can, in principle, be intentionally distorted for political reasons (both by those supporting the protests and by those opposing it) in an attempt to manipulate the public opinion (cf. “fake news”). Correspondingly, an important question arises as to how robust the parameter estimation in our model is to the uncertainty or inaccuracy in the data.

In order to look into this issue, we now consider the inverse problem where the data are subjected to ‘noise’, i.e., instead of the true numbers used in “The Yellow Vest Movement” and “Khabarovsk protests” sections, we consider ‘faked’ data distorted in a certain way. Specifically, to add noise to the data on the protesters number, we use the following formula:

$$\begin{aligned} f_\delta (t_k) = f(t_k)\left( 1 + \alpha _k \frac{\delta }{100}\right) , \qquad k=1,\ldots ,K, \end{aligned}$$

where $\delta$ is the level of noise in percents and $\alpha _k$ is a random variable uniformly distributed on the segment $[-1,1]$.

Next, we solve the inverse problem (6)–(10) and (19) using the noisy data. Parameter values obtained for the Yellow Vest Movement are shown in the Table 3. We notice that the parameters are recovered in a stable way: except for $\beta _1$, the values obtained using the noisy data are in a good agreement with theoretical sensitivity analysis carried out in “Sensitivity analysis” section.

**Table 3:** Parameter values recovered from ‘noisy’ (randomly distorted) data on the YVM for different level of noise (in percent), left to right for $\delta =0$, 5 and 10, respectively.

Noise, $\delta$	0	5	10
$\beta _1$	0.0194 $\times 10^{-9}$	0.0082 $\times 10^{-9}$	0.0912 $\times 10^{-9}$
$\beta _2$	0.1045 $\times 10^{-6}$	0.0455 $\times 10^{-6}$	0.0532 $\times 10^{-6}$
$\chi$	0.0163 $\times 10^{-2}$	0.0421 $\times 10^{-2}$	0.0289 $\times 10^{-2}$
*n*	1.4993	1.2321	1.1923
$C_0$	27975	25023	20084
$\delta _1$	0.0684	0.0708	0.0761
$\delta _2$	0.2167 $\times 10^{-3}$	0.3511 $\times 10^{-3}$	0.2655 $\times 10^{-3}$
$F_2$	4712.57 $\times 10^{6}$	4574.91 $\times 10^{6}$	4626.92 $\times 10^{6}$

We readily observe that the change in the recovered values due to the effect of noise is in most cases within 20–30%, which indicates that our procedure of parameter estimation (the inverse problem) is sufficiently robust to data uncertainty.

**Figure 14:**
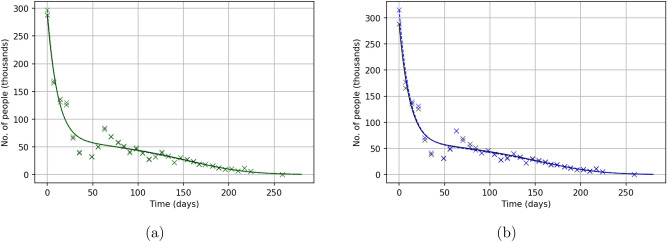
Solutions of the model (6)–(9) shown in comparison with the data on the Yellow Vest Movement for a different noise level, (**a**) for $\delta = 5\%$ and (**b**) for $\delta = 10\%$. In both cases, the black crosses show the original (undisturbed) data and the coloured crosses (green in (**a**) and blue in (**b**)) show the noisy (randomly disturbed) data. The dashed black curve (that in both cases lays very close to the coloured curve) shows the solution obtained for parameters restored from the true (undisturbed) data (cf. the left column and $\mathbf{{q}}^{(2)}$ in Table 1).

Figure 14 shows the solutions of our model obtained for parameter values restored from the noisy data as well as from the true data. We notice that, even in case of a larger noise level of 10%, the ‘noisy’ and the precise solutions (solid and dashed curves, respectively) lay very close to each other.

**Figure 15:**
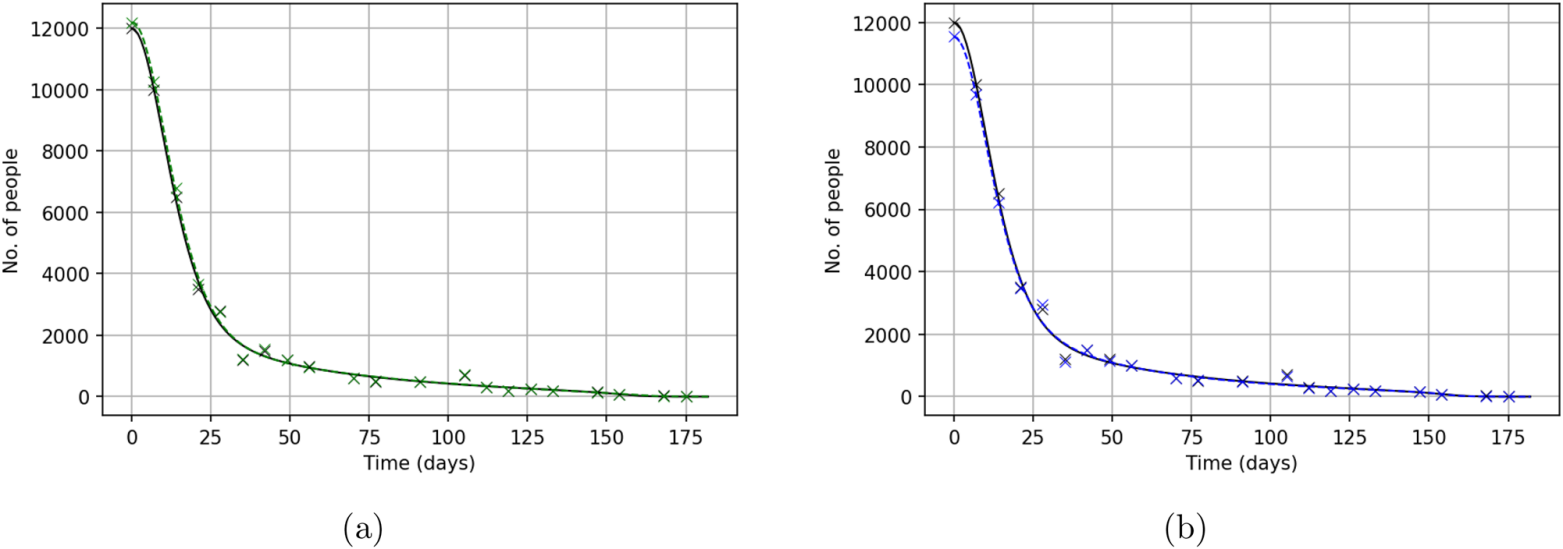
Solutions of the model (6)–(9) shown in comparison with the data on the Khabarovsk protests for a different noise level, (**a**) for $\delta = 5\%$ and (**b**) for $\delta = 10\%$. In both cases, the black crosses show the original (undisturbed) data and the coloured crosses (green in (**a**) and blue in (**b**)) show the noisy (randomly disturbed) data. The dashed black curve (that lays very close to the coloured curve) shows the solution obtained for parameters restored from the true (undisturbed) data (cf. the left column of this table and $\mathbf{{q}}^{(2)}$ in Table 2).

We now repeat the procedure using the noisy data on the Khabarovsk protests. The recovered parameters are shown in the Table 4. We observe that, in most cases, the change in parameter values due to the effect of noise is within 20–30%, except for $\beta _2$ where the change is about fivefold. The corresponding solutions of the model are shown in Fig. 15; it is readily seen that the disturbed and undisturbed solutions (dashed and solid curves, respectively) lay very close to each other providing equally good description of the data.

**Table 4:** Parameter values recovered from ‘noisy’ (randomly distorted) data on the Khabarovsk protests for different level of noise (in percent), left to right for $\delta =0$, 5 and 10, respectively.

Noise, $\delta$	0	5	10
$\beta _1$	0.1167 $\times 10^{-5}$	0.1155 $\times 10^{-5}$	0.121 $\times 10^{-5}$
$\beta _2$	0.2348 $\times 10^{-3}$	0.0484 $\times 10^{-3}$	0.2371 $\times 10^{-3}$
$\chi$	0.1408 $\times 10^{-4}$	0.6107 $\times 10^{-4}$	0.1632 $\times 10^{-4}$
*n*	3.6982	2.4061	5.8305
$C_0$	75.5561	47.3159	97.1463
$\delta _1$	0.7044	0.6677	0.7159
$\delta _2$	0.022	0.0198	0.0243
$F_2$	688192	639492	870867

Note that the change in the recovered values due to the effect of noise is in most cases within 20–30%, indicating that parameter estimation (the inverse problem) is sufficiently robust to data uncertainty.

## Discussion

Development of process-based modelling approaches to a broad variety of problems arising in social and societal dynamics, especially those linked to human behaviour, has been a fast growing theme^55^ and the new term “social physics” has been coined^56^. It was demonstrated that application of mathematical modelling can greatly improve our understanding of the corresponding social phenomena and, in case of adverse events, has a significant potential to control them in order to minimize losses and disruption and, ultimately, to save human lives^55,56^.

As one important example of this kind of problem, understanding dynamics of street protests, as well as riots and social unrest more generally, has long been a focus of research^9,32,33,39^. Over the last decade, it has been attracting a growing attention^6,16,31,38^ motivated by events such as London riots 2011, the Arab Spring, the Yellow Vest Movement, Extinction Rebellion, and others. Mathematical modelling has been shown to be an efficient tool to reveal typical patterns in the protests dynamics and identify factors and processes that affect the intensity and duration of the unrest^10,24,25,57^.

In this paper, we have focused on two recent events of street protests, namely, the Yellow Vests Movement (YVM) and the Khabarovsk protests. We mention here that, while there is considerable literature on the YVM (e.g. see^41,58^ and further references therein), including some attempts to model its dynamics^16^, there is much less literature on the Khabarovsk protests and, to the best of our knowledge, there have been no quantitative analysis or modelling studies at all. Thus, our analysis of this event appears to be the first of is kind. Our approach to combine these two cases into a single study stems from the observation that, in both cases, the curve describing protests intensity—i.e. the number of protesters vs time—exhibits a similar shape, i.e., to show an approximately monotonous decay from a large initial number to small numbers and eventually to zero. We mention here that, if considered in a broader context, this is not the only possible pattern, as there are other cases where the protests intensity curve shows a qualitatively different behaviour, e.g. the number of protesters oscillating with time^6,57^. However, arguably, the apparent similarity between the dynamics of the YVM and Khabarovsk protests suggests a certain generality of the underlying mechanism(s) regardless the significant difference between the social, cultural and political environment in France and Russia.

We assumed that the protests development and evolution through time result from the interaction between a few generic constituting elements or groups, into which the whole population can be sorted (cf. Fig. 2). In order to describe the protests quantitatively, being motivated by some previous research^9,23,25^, we used an epidemiological-type mathematical model of protests dynamics; see Eqs. (6)–(9). Essentially, this type of model describes how the human behaviour changes as a result of a social communication between different groups. We mention here that epidemiological-type models of human behaviour have been successfully used for a few different problems, e.g.^35,36^. In the context of this paper, the change in behaviour means the decision to join or leave the street protests.

We first investigated the properties of the model to show that, in a certain parameter range, it has three steady states, namely, two stable nodes and a saddle. In this range the system is bistable: to which of the two steady states system’s dynamics converge depends on the initial conditions. At the boundaries of the bistability parameter range, two of the three steady states merge and disappear as a result of the saddle-node bifurcation. Correspondingly, outside of that parameter range, there is a unique, globally stable steady state. Which of the two steady state disappears (i.e. the “upper” or the “lower”) depends on the boundary of the bistable range, cf. the left-hand side and the right-hand side parts of the panels in Fig. 3. The structure of the phase space of the model determines the properties of its solutions. Depending on parameter values, the number of protesters can show a nontrivial dependence on time (see Fig. 5). While in some cases the solution exhibits a fast, straightforward decay to zero (e.g. see the black curve in Fig. 5a), for other parameter values the dynamics of the protesters number has a few stages. As one scenario, the fast initial decay may significantly slow down at an intermediate time, so that the number of protesters would show only small changes over a relatively long time (cf. the middle part of the blue curve in Fig. 5a). In terms of the phase space structure, this critical slowing down—the ‘long transient dynamics’^17,18^—occurs when the system trajectory comes to a close vicinity of the saddle. The period of slow dynamics eventually turns into a fast decay that brings the system to its final, asymptotic state. Interestingly, the final state may be non-trivial, having a non-zero number of protesters.

We then applied the model to the two case studies of YVM and Khabarovsk. By solving the inverse problem, we obtained parameter sets that ensure the best fit of the data and showed that the agreement between the data and the corresponding solutions of the model is indeed quite good. This, in principle, opens a possibility of predicting the protests development over time.

Our modelling approach allows us to look into some ‘hidden’ properties of the protests dynamics that otherwise cannot be seen in the available data, e.g. how the number of protesters in a particular groups (i.e. novice vs mature) changes with time. It also allows us to reveal important behavioural differences in the group dynamics between the YVM and the Khabarovsk protests. In particular, we obtained that the value of parameter *n* is significantly different between the two cases (cf. Tables 1, 2): $n\approx 1.5$ for the YVM but has much larger values (in the range $3.7<n<9.5$) for the Khabarovsk protests. Here we recall that, firstly, *n* determines the number of steady states in the system (so that the bistability disappears when *n* becomes sufficiently small, e.g. see Figs. 8, 9). Secondly, and perhaps more importantly, *n* quantifies the strength of the collective response of mature protesters, in particular the degree of uniformity in the distribution of the individual decision-making threshold (see Supplementary Material). While small values of *n* correspond to a case where the threshold differs greatly between different protesters, large *n* means that the threshold is approximately the same for all mature protesters. Arguably, that can be linked to more generic social processes and structures, e.g. indicating how culturally diverse is the corresponding society. A more detailed investigation into these relations should become a focus of future research.

Our study leaves a few open questions. In particular, our analysis of the inverse problem revealed that identifiability of the model parameters is made challenging and sometimes unstable by certain discrepancy between the model structure (where the numbers of amateur and mature protesters are described by separate variables, i.e. *I* and *C*, respectively) and the nature of the data where only the cumulative protesters number $(I+C)$ is available. This discrepancy may in principle be resolved in several different ways, for instance by gaining access to more structured data that would contain some information about protesters’ characteristics, or by modifying the model (e.g. to merge the two variables together) to ensure better stability of the inverse problem with respect to data. These issues will be addressed in future research.

In conclusion, we mention that, as in any modelling study, our model takes into account some factors and processes but leaves others out of scope. Waves of contention can be quite complex^38,39^. This complexity is rooted in the complexity of the modern society, in particular in its cultural diversity and social heterogeneity, as the individual behaviours and decision making (e.g. to join or not to join the street protests) can be significantly affected by the person’s social identity^41^. Additionally, the presence of ‘counter movements’ can alter behaviour too^38^. Moreover, involvement of various social organisations may even call into question where one movement ends and the next begins. Institutionalised response to protests, e.g. through police actions, is another important factor^39^. More complex models are needed in order to investigate protests dynamics in detail. We believe that our study is the first necessary step in this long journey.

## Supplementary Information

Supplementary Information.

## Data Availability

The data on Khabarovsk street protests used in this study are freely available at: Qualitative Data Repository, QDR Main Collection, 10.5064/F6QS68PH.

## Code availability

Available computer codes used in this study can be freely accessed at: https://github.com/Aglukhov99/Dynamical-Modelling-of-Social-Protests-Yellow-Vest-Movement-and-Khabarovsk/blob/main/protests, and https://github.com/SergeiPetrovs/ProtestsModelling. Any use of the codes must be explicitly acknowledged, in particular in any publication where the simulation results obtained with this code are used.
