# Supplementary material for: Dynamical modelling of street protests using the Yellow Vest Movement and Khabarovsk as case studies
Source: Sci Rep. 2022 Nov 28;12:20447. doi: 10.1038/s41598-022-23917-z (PMC9705368; doi:10.1038/s41598-022-23917-z)
Supplement: Supplementary file 1 — Supplementary Information. [file 41598_2022_23917_MOESM1_ESM.pdf]

Dynamical Modelling of Social Protests: Yellow Vest Movement (France) and Khabarovsk (Russia) as Case Studies

Amer Alsulami, Anton Glukhov, Maxim Shishlenin, Sergei Petrovskii

Supplementary Material

Collective dynamics of mature protesters

In parameterizing the retirement rate of mature protesters, we follow the idea of Granovetter [1] about the existence of a threshold that determines decision-making: a person will make a decision to change its 'status' (e.g. their opinion or behaviour) only if a certain number - say, X - of other people in his social group have already changed their status [1]. In the context of our study, such decision means to leave the protests. Generally speaking, every individual has its own threshold, which means that any given group is described by the threshold frequency distribution - say, $f(X)$. Assuming for the sake of simplicity that function f is unimodal, the cumulative distribution $F(X)$ is a function of sigmoid shape (Fig. 1).

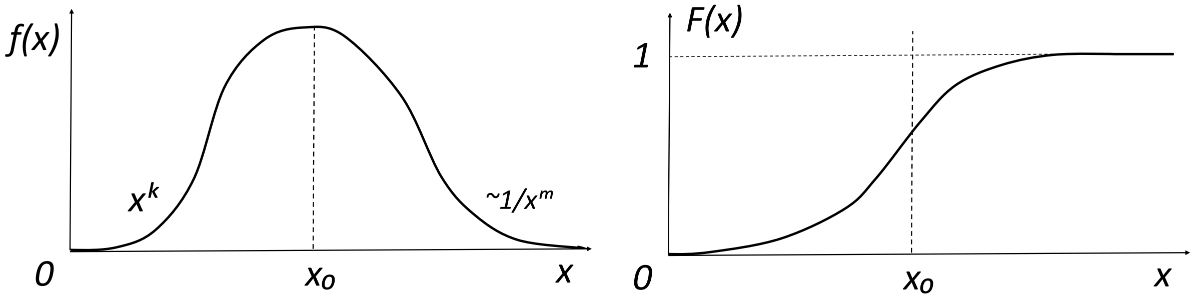

Figure 1: (a) Frequency distribution of the behavioural threshold in a social group, X_0 is the position of the maximum; (b) the corresponding cumulative distribution.

We mention here that the original Granovetter model considers the riots dynamics in a discrete time, i.e. if $X(t)$ is the number of protesters at time t, then their number at time $t+1$ is

$$X(t+1) \quad = \quad F(X(t)). \tag{1}$$

Generalization of Eq. (1) onto the continuous time is straightforward, resulting in

$$\frac{dX(t)}{dt} \quad = \quad F(X) - X. \tag{2}$$

Granovetter [1] considered $f(x)$ as a normal distribution (in which case $F(x)$ is obviously the error function). Given that the normal distribution arises, due to the Central Limit Theorem, as the asymptotic distribution for the mean of *independent* random variables (each of them having the same mean and variance), its use in the above context implicitly assumes that the formation of each individual threshold is a process entirely independent from other people in the group. This, however, is hardly realistic, as social communications affect individual behaviours virtually at any time or stage of individual development. Individual behaviours are therefore more likely to be correlated rather than independent, at least to some extent. In a system with correlations, a power law distribution (e.g. Pareto) is believed to be more relevant [4] and indeed there are many systems and processes of different origin, including economic, demographic and social ones, where factors and variables are descried by a power law [2, 3]. Correspondingly, we consider the frequency distribution $f(x)$ that has a power law asymptotics at each of its two tails (see Fig. 1a).

For the sake of simplicity, in this paper we consider a special case where the two exponents are related as $k = n - 1$ and $m = n + 1$. A cumulative distribution then can be chosen as

$$F(X) \;=\; \frac{X^n}{X^n + X_0^n}, \tag{3}$$

where a and x_0 are parameters of the distribution. Note that F is the cumulative distribution function describing the growth in the frequency of the opinion to *leave* the protests; correspondingly, the rate of change $\tilde{F}$ in the number of *remaining* protesters is $1 - F$:

$$\tilde{F}(X) \;=\; 1 - F \;=\; \frac{X_0^n}{X^n + X_0^n}. \tag{4}$$

Equation (4) determines the structure of the right-hand side of Eq. (5) in the main text.

References

[1] Granovetter M (1978). Threshold models of collective behavior. American Journal of Sociology 83, 1420-1443.

[2] Newman MEJ (2005) Power laws, Pareto distributions and Zipf's law. Contemporary Physics 46, 323351.

[3] Reed WJ, Jorgensen M (2004) The double Pareto-lognormal distribution - a new parametric model for size distributions. Communications in Statistics – Theory and Methods 33, 1733-1753.

[4] Sornette D (2000) Critical Phenomena in Natural Sciences: Chaos, Fractals, Self Organization and Disorder: Concepts and Tools. Springer, Berlin.
